# A novel myogenic function residing in the 5′ non-coding region of *Insulin receptor substrate-1* (*Irs-1*) transcript

**DOI:** 10.1186/s12860-015-0054-8

**Published:** 2015-03-11

**Authors:** Hikaru Nagano, Naoko Yamagishi, Chisato Tomida, Chiaki Yano, Kana Aibara, Shohei Kohno, Tomoki Abe, Ayako Ohno, Katsuya Hirasaka, Yuushi Okumura, Edward M Mills, Takeshi Nikawa, Shigetada Teshima-Kondo

**Affiliations:** Department of Nutritional Physiology, Institute of Health Biosciences, Tokushima University Graduate School, Tokushima, 770-8503 Japan; Graduate school of Fisheries Science and Environmental Studies, Nagasaki University, Nagasaki, 852-8521 Japan; Facalty of Nutritional Science, Sagami Women’s University, Sagamihara, 252-0383 Japan; Division of Pharmacology/Toxicology, College of Pharmacy, University of Texas at Austin, Austin, Texas

**Keywords:** Bifunctional RNA, Insulin receptor substrate-1, Myogenic differentiation, Rb

## Abstract

**Background:**

There is evidence that several messenger RNAs (mRNAs) are bifunctional RNAs, *i.e.* RNA transcript carrying both protein-coding capacity and activity as functional non-coding RNA via 5′ and 3′ untranslated regions (UTRs).

**Results:**

In this study, we identified a novel bifunctional RNA that is transcribed from insulin receptor substrate-1 (*Irs-1*) gene with full-length 5′UTR sequence (FL-*Irs-1* mRNA). FL-*Irs-1* mRNA was highly expressed only in skeletal muscle tissue. In cultured skeletal muscle C2C12 cells, the FL-*Irs-1* transcript functioned as a bifunctional mRNA. The FL-*Irs-1* transcript produced IRS-1 protein during differentiation of myoblasts into myotubes; however, this transcript functioned as a regulatory RNA in proliferating myoblasts. The FL-*Irs-1* 5′UTR contains a partial complementary sequence to Rb mRNA, which is a critical factor for myogenic differentiation. The overexpression of the 5′UTR markedly reduced Rb mRNA expression, and this reduction was fully dependent on the complementary element and was not compensated by IRS-1 protein. Conversely, knockdown of FL-*Irs-1* mRNA increased Rb mRNA expression and enhanced myoblast differentiation into myotubes.

**Conclusions:**

Our findings suggest that the FL-*Irs-1* transcript regulates myogenic differentiation as a regulatory RNA in myoblasts.

**Electronic supplementary material:**

The online version of this article (doi:10.1186/s12860-015-0054-8) contains supplementary material, which is available to authorized users.

## Background

Recent genome-wide transcriptome studies discovered that the mammalian genome is pervasively transcribed into RNA. Within the protein-coding gene regions, there are many alternative transcriptional start sites, which results in the production of not only protein-coding mRNA but also non-protein-coding RNAs (ncRNAs) [1-6].

Intriguingly, the recent identification and characterization of various mRNAs demonstrated that there are several bifunctional mRNAs. The bifunctional RNA transcripts have both protein-coding capacity and activity as functional ncRNA in a context-dependent manner [7-9]. For example, steroid receptor activator RNA (SRA) acts as bifunctional transcript. Non-coding SRA stimulates transcriptional activity of MyoD and enhances muscle cell differentiation, whereas SRA protein binds to and counteracts SRA ncRNA function [10-13]. p53 mRNA interacts with MDM2 protein and prevents MDM2 function [14]. The untranslated region (UTR) of several mRNA transcripts can also function as a trans-acting regulatory RNA that is independent of the protein-coding functions. Several mRNA 3′UTRs act as competing endogenous RNAs (ceRNAs) or natural microRNA sponges. Examples include the 3′UTR of PTEN mRNA [15], the 3′UTR of ZEB2 mRNA [16] and the 3′UTR of Hmga2 mRNA [17]. The 5′UTR of mRNA also acts as regulatory RNA, as we have previously reported that the 5′UTR of *VEGF* mRNA promotes tumor malignancy by silencing of STAT1 mRNA expression [18].

Skeletal muscle differentiation is a powerful system for investigating functional RNAs, because it can be recapitulated *in vitro* and because the myogenic differentiation program is well characterized and evolutionarily conserved [19]. There is increasing evidence to support that several ncRNAs, such as linc-MD1, SINE-containing ncRNAs, and the 3′UTR of DMPK (dystrophia myotonica protein kinase) mRNA regulate myogenesis through modulation of myogenic gene expression, such as Pax3, MyoD, Myf5, and miRNA-133 and −135 [20-23].

Skeletal muscle differentiation occurs by a stepwise progression. The expression of the retinoblastoma protein (Rb) is critically important to this process [24-30]. The role of Rb is multifaceted and includes the orchestration of cell cycle arrest and prevention of cell cycle reentry. Furthermore, Rb also enforces a stable muscle-specific gene expression. Thus, the silencing of Rb expression by siRNA or gene knockout abrogates myoblast differentiation [25,28].

In this study, we focused on insulin receptor substrate-1 (*Irs-1*) gene products because IRS-1 plays critical roles in myogenic growth and differentiation as previously demonstrated by our group and others [31,32]. We found that one of the transcriptional variants of *Irs-1* mRNA was highly expressed in skeletal muscle relative to liver and adipose tissue. We demonstrate that the *Irs-1* transcript is a bifunctional mRNA. The 5′UTR of the *Irs-1* transcript repressed Rb mRNA expression and suppressed muscle cell differentiation. Our results suggest that the *Irs-1* transcript is a regulator of myogenic differentiation.

## Results

### Characterization of mouse *Irs-1* transcriptional variants

**Figure 1:**
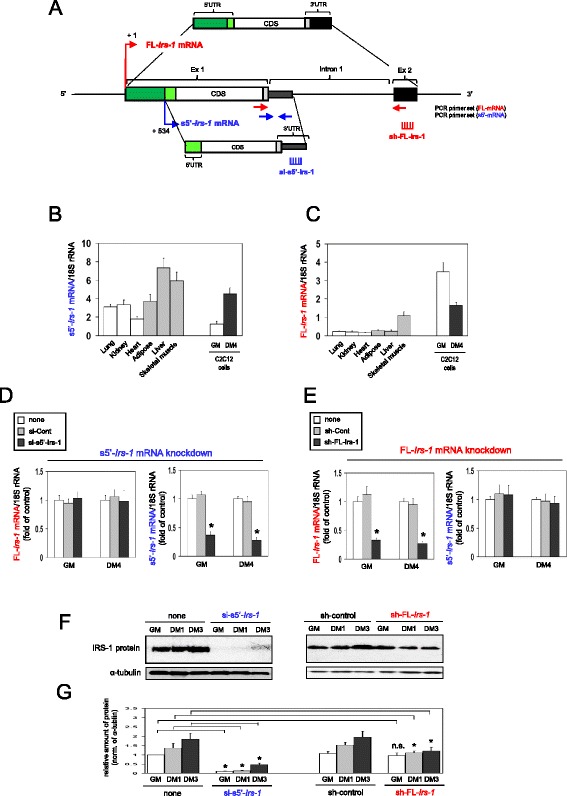
**Characterization of two distinct** ***Irs-1*** **transcripts. (A)** Schematic representation of two transcriptional variants of mouse *Irs-1* mRNAs (FL-*Irs-1* and s5′-*Irs-1* mRNAs). The positions of specific qPCR primer sets are shown as arrows (red is for FL-*Irs-1*, blue is for s5′-*Irs-1* mRNA). **(B)** Expression levels of s5′-*Irs-1* mRNA in the indicated mouse tissues, proliferating C2C12 myoblasts (GM, growth medium) and differentiating C2C12 myotubes (DM4, differentiation medium for 4 days). Mean ± SD, n = 4-6. **(C)** Expression levels of FL-*Irs-1* mRNA in the indicated mouse tissues, C2C12 myoblasts (GM) and C2C12 myotubes (DM4). Mean ± SD, n = 4-6. **(D,E)** Specific knockdown of FL-*Irs-1* mRNA (d) or s5′-*Irs-1* mRNA (e) in myoblasts (GM) and myotubes (DM4). Mean ± SD, n = 4, *p < 0.01 *versus* no treatment (none). **(F)** Effect of specific knockdown of FL-*Irs-1* mRNA or s5′ *Irs-1* mRNA on IRS-1 protein expression during myogenesis. **(G)** Quantification analysis and statistic were performed on three independent experiments. Mean ± SD, *p < 0.01 *versus* each culture conditions (GM, DM1 or DM3) in no transfection (none). n.s., not significant.

Mouse *Irs-1* gene consists of two exons and one intron. The exon 1 encodes 5′UTR, IRS-1 protein sequence and partial 3'UTR, while the exon 2 encodes only 3′UTR (Figure 1A). A bioinformatics analysis of the mouse *Irs-1* gene region using the UCSC genome database [33] revealed 11 different transcriptional variants in the *Irs-1* gene region, including IRS-1 protein-coding mRNAs and non-protein coding transcripts. Each of the transcripts is derived from a distinct transcription start site and has a transcriptional termination site. The database includes tissue- and cell type-specific expression of *Irs-1*-related transcripts (Additional file 1: Figure S1A). For instance, heart and liver express *Irs-1* mRNA (GenBank accession number AK045317), while brain poorly expresses it. Fibrocytes and fibroblasts express several *Irs-1* transcripts, whereas MEL cells, erythroblasts and megakaryocytes do not express any *Irs-1* transcripts. Notably, exon 2-derived transcripts (BC034138, AK052241, AK136875, AK141842 and AK169784) that do not encode IRS-1 protein are highly expressed in several tissues (cerebellum, brain, heart and liver) and cells (fibrocytes and fibroblasts). Fibroblasts clearly express intron 1-derived non-coding transcript (AK137314) (Additional file 1: Figure S1A).

We tried to measure the expression profile of these unique transcripts in several mouse tissues and C2C12 muscle cell lines by quantitative RT-PCR (qRT-PCR) using specific primer sets for each transcript (indicated in Additional file 1: Figure S1A). The intron 1-derived transcript (AK137314) was ubiquitously expressed (Additional file 1: Figure S1B). However, we could not determine the levels of exon 2-derived transcripts (BC034138, AK052241, AK136875 and AK141842), since they were not amplified by qPCR using our primer set.

### Characterization of two distinct *Irs-1* mRNAs expression

We are interested in whether the 5′UTR of mRNA acts as functional RNA similar to results we previously demonstrated [18]. Therefore, we focused on two *Irs-1* mRNA variants that have different 5′UTRs because of different transcription start sites (Figure 1A). As shown in Figure 1A, one is transcribed from a + 1 nucleotide (nt) transcription start site in exon 1, thus it contains full-length 5′UTR sequence (FL-*Irs-1* mRNA). The other variant is transcribed from a + 534 nt transcription start site in exon 1 and results in a short 5′UTR sequence (s5′-*Irs-1* mRNA). Both FL- and s5′-*Irs-1* mRNAs contain the same protein coding sequence (CDS) encoding exon 1 and should produce the same IRS-1 protein (Figure 1A).

We then examined the expression profile of the two *Irs-1* transcripts in several mouse tissues by qRT-PCR using a specific primer set for each transcript (indicated in Figure 1A). s5′-*Irs-1* mRNA was ubiquitously expressed in multiple tissues, especially in adipose tissue, liver and skeletal muscle that are well known tissues highly expressing IRS-1 protein [34-36] (Figure 1B). Interestingly, FL-*Irs-1* mRNA was highly expressed only in skeletal muscle (Figure 1C), which suggests that FL-*Irs-1* mRNA may have a skeletal muscle-specific function.

To determine the expression profile of the two *Irs-1* mRNA transcripts during myogenesis, we used mouse myoblast cell line C2C12 that is a well characterized model system for studying muscle differentiation *in vitro*. C2C12 cells proliferate as myoblasts in growth medium (GM), while confluent C2C12 myoblasts exit the cell cycle and fuse with one another to form multinucleated muscle cells (myotubes) in differentiation medium (DM). The expression levels of s5′-*Irs-1* mRNA were low in myoblasts and increased by 3.6-fold in myotubes (Figure 1B). In contrast, FL- *Irs-1* mRNA levels were relatively high in myoblasts, but decreased in differentiated myotubes (Figure 1C). When comparing the levels of s5′- and FL-*Irs-1* mRNAs between skeletal muscle tissue and C2C12 myotubes, expression levels of both mRNAs were similar (Figure 1B,C). In C2C12 myoblasts, s5′-*Irs-1* mRNA levels were lower than skeletal muscle tissue, while FL-*Irs-1* mRNA levels were higher (Figure 1B,C).

To determine an expression relationship between the two *Irs-1* mRNAs and IRS-1 protein in C2C12 cell myogenic process, FL-*Irs-1* mRNA or s5′-*Irs-1* mRNA was specifically knocked down by targeting their 3′UTR sequence encoded in exon 2 (FL-*Irs-1* mRNA) or in intron 1 (s5′-*Irs-1* mRNA) (Figure 1A). The si-s5′-*Irs-1* reduced s5′-*Irs-1* mRNA levels to approximately 30% of control cells without affecting FL-*Irs-1* mRNA levels (Figure 1D). The sh-FL-*Irs-1* efficiently decreased FL- *Irs-1* mRNA levels to approximately 20% compared with the sh-control and did not alter s5′-*Irs-1* mRNA levels (Figure 1E).

In untransfected (none) or control transfected cells (sh-control), the IRS-1 protein levels were increased during differentiation from myoblasts to myotubes (Figure 1 F,G). When FL-*Irs-1* mRNA was knocked down, the IRS-1 protein levels decreased in differentiating myotubes but not in proliferating myoblasts (Figure 1 F,G). In contrast, knockdown of s5′-*Irs-1* mRNA clearly reduced IRS-1 protein levels in both myoblasts and myotubes (Figure 1 F,G). These results indicate that the s5′-*Irs-1* transcript participates in continuous IRS-1 protein production during myogenesis and that FL-*Irs-1* mRNA is involved in IRS-1 protein synthesis only in differentiating myotubes.

### FL-*Irs-1* transcript regulates myoblast differentiation

**Figure 2:**
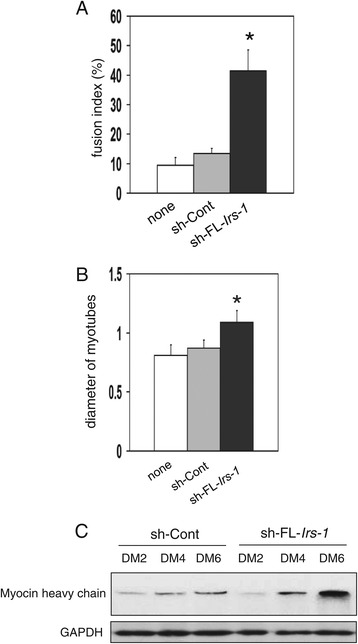
**FL-** ***Irs-1*** **transcript regulates C2C12 differentiation. (A,B)** Effect of knockdown of FL-*Irs-1* mRNA on differentiation of C2C12 myoblast. Fusion index **(A)** and a mean diameter of myotubes **(B)** are shown. Mean ± SD, n = 50, *p < 0.01 *versus* no treatment (none). **(C)** Effect of knockdown of FL- *Irs-1* mRNA on expression levels of myosin heavy chain in differentiating C2C12 cells (DM2, 4 and 6).

In myoblasts, the FL-*Irs-1* transcript was highly expressed and was not involved in IRS-1 protein production. Therefore, we hypothesized that FL-*Irs-1* mRNA functions as an ncRNA with activity independent of its protein-coding function. To address this hypothesis, we examined the effect of knockdown of FL-*Irs-1* transcript on the C2C12 cell phenotype. The knockdown of FL- *Irs-1* mRNA markedly enhanced differentiation into myotubes (Figure 2). The results of fusion index show that the number of differentiated myotubes was increased more than 3-fold by knockdown of FL- *Irs-1* mRNA compared with untransfected and sh-control-transfected cells (Figure 2A). The mean diameter of myotubes was also increased by approximately 130% after knockdown of FL-*Irs-1* mRNA (Figure 2B). The expression of myosin heavy chain, which is a myogenic differentiation marker, was also enhanced by knockdown of FL-*Irs-1* mRNA (Figure 2C). In contrast, knockdown of s5′-*Irs-1* mRNA did not affect C2C12 cell differentiation (Additional file 2: Figure S2). These data indicate that accelerated differentiation by knockdown of FL-*Irs-1* transcript was not caused by a reduction of IRS-1 protein, but instead, by a blockade of FL-*Irs-1* transcript function.

It is well known that Rb plays a critical role in inducing myogenic differentiation, and the introduction of Rb in myoblasts accelerates differentiation to myotubes [24-26]. Conversely, inhibition of Rb expression in myoblasts abrogates differentiation into myotubes [27,28]. Thus, we hypothesized that FL-*Irs-1* transcript may affect Rb expression.

### FL*-Irs-1* 5′UTR reduces Rb mRNA expression in C2C12 cells

**Figure 3:**
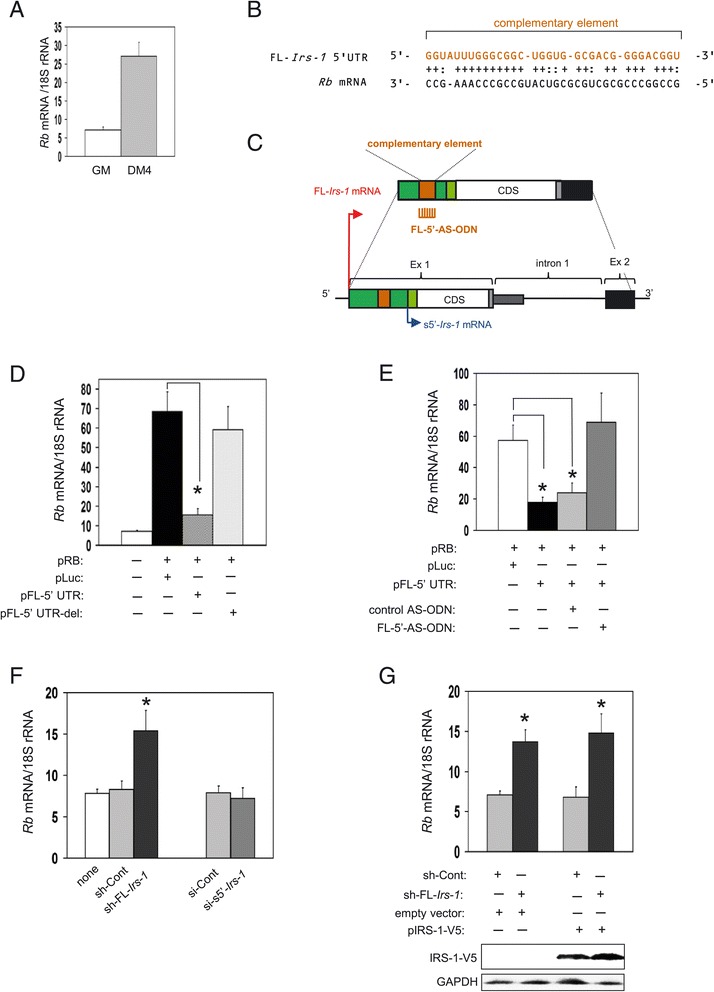
**FL-** ***Irs-1*** **transcript reduces Rb mRNA levels. (A)** Expression levels of Rb mRNA in C2C12 myoblasts (GM) and myotubes (DM4). **(B)** Partial sequence complementarity between the 5′UTR of FL-*Irs-1* transcript (nt 373 – nt 405) and Rb mRNA (nt 131 – nt 165). **(C)** Schematic representation of position of complementary element in the 5′UTR of FL-*Irs-1* transcript. A position of an LNA-based antisense oligonucleotide (FL-5′-AS-ODN) is shown. **(D)** Effect of FL-*Irs-1* 5′UTR on Rb mRNA expression levels. C2C12 myoblasts were untransfected (−) or transfected with an Rb mRNA-expressing plasmid in combination with the indicated effector plasmid (pFL-5′UTR, pFL-5′UTR-del). Rb mRNA levels were measured by qRT-PCR. Mean ± SD, n = 4-5, *p < 0.01 *versus* control transfectant (pRB + pLuc). **(E)** Effect of FL-5′-AS-ODN on Rb mRNA reduction by FL-*Irs-1* 5′UTR. C2C12 myoblasts were co-transfected with plasmids expressing Rb mRNA and the FL-*Irs-1* 5′UTR RNA in combination with the indicated AS-ODN. Mean ± SD, n = 4-5, *p < 0.01 *versus* control transfectant (pRB + pLuc). **(F)** Effect of knockdown of endogenous FL-*Irs-1* transcript on endogenous Rb mRNA levels. C2C12 myoblasts were transfected with the indicated shRNA or siRNA. Mean ± SD, n = 4, *p < 0.01 *versus* no treatment (none). **(G)** Effect of supplementary expression of IRS-1 protein on Rb mRNA levels under FL-*Irs-1* transcript knockdown conditions. C2C12 myoblasts were transfected with the sh-FL-*Irs-1* plasmid in combination with a plasmid encoding IRS-1 protein fused to V5 tag (pIRS-1-V5). Expression levels of exogenous IRS-1 protein were determined by immunoblot analysis using anti-V5 antibody. Mean ± SD, n = 3, *p < 0.01 *versus* control transfectant (sh-Control).

We examined the expression levels of Rb mRNA in myoblasts and myotubes. Myoblasts expressed low levels of Rb mRNA and myotubes expressed higher levels (Figure 3A). The Rb mRNA expression profile had an inverse relationship with FL-*Irs-1* transcript levels (Figure 1C, C2C12 cells).

A bioinformatics analysis showed that there was a partial sequence-complementary element between the 5′UTR of FL-*Irs-1* transcript and Rb mRNA (Figure 3B,C). Given that several functional RNA transcripts can interact with target mRNAs by intermolecular base-pairing via a partially complementary element [21,22,37], FL-*Irs-1* 5′UTR may target Rb mRNA via the complementary element. To assess whether FL- *Irs-1* 5′UTR participated in Rb expression, C2C12 myoblasts were transfected with an Rb mRNA-expressing plasmid in combination with a plasmid expressing the FL-*Irs-1* 5′UTR fused to the luciferase gene (pFL-5′UTR-Luc) or a control luciferase plasmid (pLuc). Overexpression of the 5′UTR, but not control plasmid, markedly reduced Rb mRNA levels (Figure 3D). To demonstrate that the complementary element in the 5′UTR was responsible for the reduction of Rb mRNA, we deleted the complementary element (pFL-5′UTR-del). The deletion of the element fully abrogated the ability to decrease Rb mRNA expression (Figure 3D).

To exclude the possibility that the deletion may affect the secondary structure of FL-*Irs-1* 5′UTR, resulting in a loss of silencing activity for Rb mRNA, we examined effect of an LNA-based antisense oligonucleotide against the complementary element (FL-5′-AS-ODN). FL-5′-AS-ODN, but not control AS-ODN, completely prevented the decrease of Rb mRNA expression induced by FL-*Irs-1* 5′UTR (Figure 3E). These results indicated that FL-*Irs-1* 5′UTR decreased Rb mRNA levels via its complementary sequence.

To verify that endogenous FL-*Irs-1* transcript participated in endogenous Rb mRNA expression, we knocked down the FL-*Irs-1* transcript. The silencing of FL-*Irs-1* mRNA increased Rb mRNA levels by more than 2-fold (Figure 3 F). In contrast, knockdown of s5′-*Irs-1* mRNA did not affect the Rb mRNA levels. These results suggest that the elevation of Rb mRNA levels was not caused by decreasing IRS-1 protein after knockdown of FL-*Irs-1* transcript. The supplementary expression of exogenous IRS-1 protein using an IRS-1-expressing plasmid that encodes only the open reading frame of IRS-1 without the 5′ and 3′-UTRs did not affect the elevation of Rb mRNA levels (Figure 3G).

We additionally examined the effect of knockdown of FL-*Irs-1* transcript on other myogenic regulators expression, such as MyoD, myogenin, Myf5, MRF4 and MEF2. The silencing of FL-*Irs-1* mRNA did not affect the levels of all myogenic factors tested (Additional file 3: Figure S3).

### FL-*Irs-1* 5′UTR does not reduce Rb mRNA expression in hepatocytes

**Figure 4:**
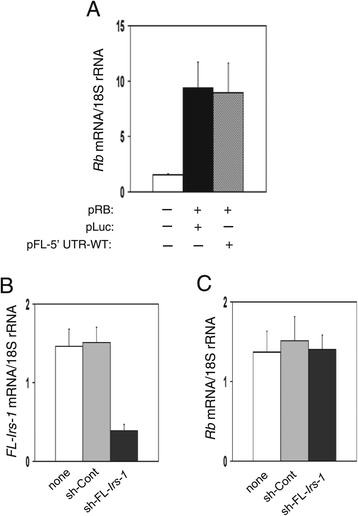
**FL-** ***Irs-1*** **transcript does not reduce Rb mRNA expression in hepatocytes. (A)** Effect of FL-*Irs-1* 5′UTR on Rb mRNA expression levels. Cultured hepatocyte cell lines (NCTC clone 1469) were untransfected (−) or transfected with an Rb mRNA-expressing plasmid in combination with the indicated effector plasmid. Rb mRNA levels were measured by qRT-PCR. Mean ± SD, n = 4, *p < 0.01 *versus* control transfectant (pRB + pLuc). **(B,C)** Effect of knockdown of endogenous FL-*Irs-1* transcript on endogenous Rb mRNA levels. Cultured hepatocyte cell lines were transfected with the indicated shRNA. FL-*Irs-1* **(B)** and Rb mRNA **(C)** levels were measured by qRT-PCR. Mean ± SD, n = 4, *p < 0.01 *versus* no treatment (none).

We further tested whether the FL-*Irs-1* 5′UTR-induced Rb mRNA reduction occurred in hepatocytes because IRS-1 is an important factor in hepatocytes. A cultured hepatocyte cell line was co-transfected with plasmids expressing Rb mRNA and the FL-*Irs-1* 5′UTR. Unlike skeletal muscle cells, the overexpression of the 5′UTR did not reduce Rb mRNA levels in hepatocytes (Figure 4A). In addition, knockdown of FL-*Irs-1* transcript did not alter Rb mRNA levels in hepatocytes (Figure 4B,C).

### Reduction of Rb mRNA by FL-*Irs-1* transcript did not require Dicer and Upf1

We then examined the molecular mechanism responsible for FL-*Irs-1* 5′UTR reducing Rb mRNA levels. There is evidence that several miRNAs and ncRNAs regulate myogenic differentiation through the Dicer- and Upf1-mediated target mRNA decay system [21,22]. The knockdown of Dicer or Upf1 by siRNA (Additional file 4: Figure S4A, B) did not alter the suppression of Rb mRNA by FL-*Irs-1* 5′UTR (Additional file 4: Figure S4C). In addition, northern blot analysis using FL-5′-AS-ODN showed a small RNA with approximately 20–25 nt in length or the 5′UTR RNA distinct from FL-*Irs-1* mRNA was not detected in either myoblasts and myotubes (Additional file 4: Figure S4D). These data suggest that FL-*Irs-1* transcript was not processed to miRNA or intermediate 5′UTR RNA.

## Discussion

In this study, we found that FL-*Irs-1* transcript acts as a bifunctional RNA. One function is as a regulatory RNA in proliferating myoblasts that reduced Rb mRNA expression and suppressed myoblast differentiation. The other function is as mRNA that produced IRS-1 protein during differentiation into myotubes. By contrast, s5′-*Irs-1* mRNA was only involved in IRS-1 protein synthesis during myogenesis.

Rb plays a crucial role in the switch from proliferation to differentiation in myoblasts [24-28]. Myoblasts lacking Rb lose the capacity to differentiate into myotubes [27,28], and the overexpression of Rb in myoblasts enhances differentiation into myotubes [25]. It is well established that Rb function is regulated by posttranslational modification (phosphorylation levels) during myoblast differentiation. Although it is considered that the Rb expression levels are tightly regulated in myoblasts before and during differentiation, how Rb levels are regulated in myoblasts is still unclear. In this study, the knockdown of endogenous FL-*Irs-1* transcript increased Rb mRNA levels and accelerated differentiation of myoblasts to myotubes. Thus, our findings suggest that FL-*Irs-1* transcript could be one of the endogenous downregulator(s) of Rb expression in myoblasts.

A functional relevance of between IRS-1 protein and Rb protein was previously reported [38-40]. IRS-1 inhibits transcriptional activity of Rb [38]. In addition, several reports indicate that the IGF-1/IRS-1 pathway suppresses Rb-mediated cell cycle arrest by inducing prolonged phosphorylation of Rb [39,40]. However, at least to our best knowledge, our study is the first report showing a relationship between *Irs-1* transcript and Rb mRNA.

There are several ncRNAs that regulate myogenesis through modulation of myogenic gene expression, such as Pax3 [21], Myf5 [22], MyoD [23], and miRNA-133 and −135 [20]. Several SINE-containing ncRNAs and mRNAs interact with their target mRNAs such as Myf5 by base-pairing with partially complementary elements to induce degradation of them and regulate C2C12 cell myogenesis [21,22]. In these cases, the silencing of target mRNAs by the ncRNAs requires base-pairing to target mRNA and a Dicer- and Upf1-dependent mechanism [21,22]. Here, we demonstrated that the reduction of Rb mRNA by FL-*Irs-1* transcript did not require Dicer and Upf1. However, the complementary element in the 5′UTR of FL-*Irs-1* transcript was necessary to decrease Rb mRNA levels. In addition, the reduction of Rb mRNA by the FL-*Irs-1* transcript was not observed in hepatocytes despite the important role of Rb in hepatocyte differentiation [41]. Thus, skeletal muscle-specific factors may be required in Rb mRNA silencing. Further studies are required to clarify a molecular mechanism of FL-*Irs-1* transcript-mediated gene silencing.

Until recently, it has been generally accepted that mRNA functions only in protein production, and cell phenotypes depend exclusively on the function of these proteins. Unexpectedly, recent studies demonstrated that several mRNAs may have bifunctional abilities in a context-dependent manner. It is predicted that the human genome contains approximately 300 possible potentially new bifunctional mRNAs [8]. One characterized bifunctional mRNA is SRA during myogenic processes [10-13]. In myogenic proliferation, the protein-coding SRA transcript is abundantly expressed, and SRA protein binds to and counteracts SRA ncRNA-mediated MyoD activation and suppresses differentiation [10]. During myogenic differentiation, the expression of non-coding SRA transcript is elevated and enhances transcriptional activity of MyoD and differentiation [11-13]. These findings suggest that the effect of SRA on myogenesis may result from the correct balance between non-coding and coding SRA transcripts. The present study showed that the balance between coding and non-coding *Irs-1* transcripts may be important for regulating myogenic differentiation.

Beyond UTRs function as *cis* regulatory elements that impact the translational activity and stability of their own mRNAs, UTRs are also *trans* modulator of gene expression through their target transcript inhibition as demonstrated here and by others [15-17]. As recently suggested by the Pandolfi group [42-45], mRNA transcripts can potentially perturb the interaction with target RNAs and possess a biological activity independent of the translation of the protein they encode. Therefore, some of the phenotypic features of cells may depend not only on the protein function but also on the regulatory RNA function that is distinct from their protein products.

## Conclusions

The objective of this study was to identify a novel bifunctional RNA in myogenic process. We found that FL-*Irs-1* transcript regulates Rb expression at the RNA levels independent of its protein-coding function. Our findings suggest that FL-*Irs-1* transcript functions as a bifunctional RNA during myogenic differentiation.

## Methods

### Cell culture

C2C12 myoblast cells were cultured in Dulbecco’s Modified Eagle Medium (DMEM) containing 10% fetal bovine serum (FBS) and penicillin-streptomycin mixed solution (Nacalai Tesque, Kyoto, JAPAN) at 37°C with 5% CO_2_. When confluent, myogenic differentiation was induced by changing media to DMEM supplemented with 2% horse serum and penicillin-streptomycin mixed solution at 37°C with 5% CO_2_. Mouse hepatocyte cell lines (NCTC clone 1469) were cultured in NCTC 135 medium containing 10% horse serum and penicillin-streptomycin mixed solution at 37°C with 5% CO_2_.

### siRNA and transfection

Stelth RNAi negative control siRNA (medium GC content, Invitrogen) or Silencer Select negative control #1 siRNA (Ambion) was used as a control siRNA, which has no homology to human gene products. The siRNA targeting mouse Dicer mRNA duplex targets 5′-GCCGAUCUCUAAUUACGUA TT-3′ (Ambion). The siRNA targeting mouse Upf1 mRNA targets 5′-CAGUUACUGUGGAAUCCAUTT-3′ (Ambion). The siRNAs targeting s5′-Irs1 mRNA targets 5′-UCGCCAAACUUAACGUUCUAUCAAA-3′ (Invitrogen). Cells were transfected with siRNA using jetPRIME™ DNA reagent (Polyplus-transfection, Illkirch, France), according to the manufacturer’s instructions. Transfection efficiency of siRNA was approximately 60-70% that was determined using BLOCK-iT Alexa Fluor Red Fluorescent Oligo (Invitrogen).

### Plasmid construction and transfection

A plasmid encoding mouse Rb sequence was purchased from Kazusa DNA Res. Inst. A plasmid expressing IRS-1 protein was constructed as described previously [31]. FL-*Irs-1* 5′UTR and FL-*Irs-1* 5′UTR-del sequences were synthesized by custom gene synthesis technology (GenScript Inc). The synthesized double strand DNA was cloned into the phCMV-FSR luciferase vector (pLuc, Genlantis). Insert sequence was verified by DNA sequencing.

A plasmid expressing shRNA targeted against FL-Irs1 mRNA exon 2(5′- GGAGAGAGTATTAAATATT -3′) was constructed using the piGENE-U6-Rep vector (iGene Therapeutics Inc., Tsukuba, Japan). The piGENE-U6-Rep vector containing seven tandem repeats of thymidine (T7) served as the negative control vector. The shRNAs were transfected into cells by jetPRIME™ DNA reagent according to manufacturer’s instructions. Transfected cells were selected with 4 ug/ml puromycin 24 hours after transfection and changed to fresh selection medium for additional 10 days.

### Quantitative RT-PCR (qRT-PCR)

SuperScript II RNase H-reverse transcriptase (Invitrogen) was used to synthesize cDNAs from 500 ng aliquots of total RNA. The levels of s5′-Irs1 mRNA, FL-Irs1 mRNA, Rb mRNA and 18S ribosomal RNA were measured by real time (RT)-PCR using the following specific primer sets: s5′-Irs1, 5′- TATGCCAGCATCAGCTTCC -3′ (forward) and 5′- TAAAAACGCACCTGCTGTGA -3′ (reverse); FL-Irs1, 5′- CTATGCCAGCATCAGCTTCC -3′ (forward) and 5′- TTGCTGAGGTCATTTAGGTCTTC −3′ (reverse); Rb, 5′- AATTAGAACGGACGTGTGAACTT -3′ (forward) and 5′- CCAAGAAATTTTTAGCACCAACA -3′ (reverse); 18S rRNA, 5′- GCAATTATTCCCCATGAACG -3′ (forward) and 5′- GGGACTTAATCAACGCAAGC -3′ (reverse). Amplification and quantification of the PCR products were performed using the Applied Biosystems 7500 System (Applied Biosystems). Standards were run in the same plate and the relative standard curve method was used to calculate the relative mRNA expression. RNA amounts were normalized against the18S ribosomal RNA level.

### Western and Northern blot analyses

Cell lysates were prepared using a lysis buffer containing 100 mM Tris–HCl (pH 6.8), 300 mM NaCl, 2 mM EDTA and 4% (v/v) SDS. Western immunoblotting was performed as described previously [18,31] using rabbit polyclonal anti-IRS-1 (Calbiochem, La Jolla, CA), mouse monoclonal anti-MHC (myosin heavy chain, fast) (Sigma), rabbit polyclonal anti-α-tubulin (Santa Cruz Biotechnology, USA), or rabbit polyclonal anti-GAPDH (Santa Cruz Biotechnology, USA). Densitometric analysis was performed using the ImagePro software as described previously [18].

Total RNA was prepared by the guanidinium thiocyanate method using ISOGEN reagent (Wako, Japan). Northern hybridization was performed as described previously [18] using LNA-based antisense oligonucleotide probe for the complementary sequence in the FL- *Irs1* mRNA 5′UTR (Figure 3B). The hybridized blots were exposed to BAS1500 Imaging screen (FujiFilm) for 1 h, then the images were developed.

### Measurement of myotube number and diameter

Nuclei of C2C12 myotubes were fixed and stained using propidium iodide. Each well was photographed in six randomly selected regions at × 20 magnifications at day 3 or day 5 post-differentiation induction using a BIOREVO BZ-9000 fluorescent microscope (Keyence, Osaka, Japan). The number of nuclei incorporated in myotubes and the total number of nuclei were scored. Fusion index was calculated as the percentage of total nuclei incorporated in myotubes. Diameter of myotubes was measured using BIOREVO BZ-9000 software (Keyence).

### Statistical analysis

Results are expressed as means ± S.D. Statistical analyses of data were done using ANOVA and the Scheffé’s test. P values < 0.05 was considered significant.

## Additional files

Additional file 1: Figure S1. Abundance and tissue/cell type distribution of *Irs-1* transcript variants. Additional file 2: Figure S2. Effect of s5′-*Irs-1* transcript on C2C12 differentiation. Additional file 3: Figure S3. Effect of FL-*Irs-1* transcript on expression of several myogenic factors. Additional file 4: Figure S4. Effect of knockdown of Dicer or Upf-1 on FL-Irs-1 function.

## Abbreviations

IRS-1: Insulin receptor substrate-1
UTR: Untranslated region
Rb: Retinoblastoma protein
