# Supplementary material for: A novel myogenic function residing in the 5′ non-coding region of Insulin receptor substrate-1 (Irs-1) transcript
Source: BMC Cell Biol. 2015 Mar 11;16:8. doi: 10.1186/s12860-015-0054-8 (PMC4373113; doi:10.1186/s12860-015-0054-8)
Supplement: Additional file 1: Figure S1. — Abundance and tissue/cell type distribution of Irs-1 transcript variants. [file 12860_2015_54_MOESM1_ESM.pdf]

Supplementary Figure 1 (Nagano *et al.*)

A

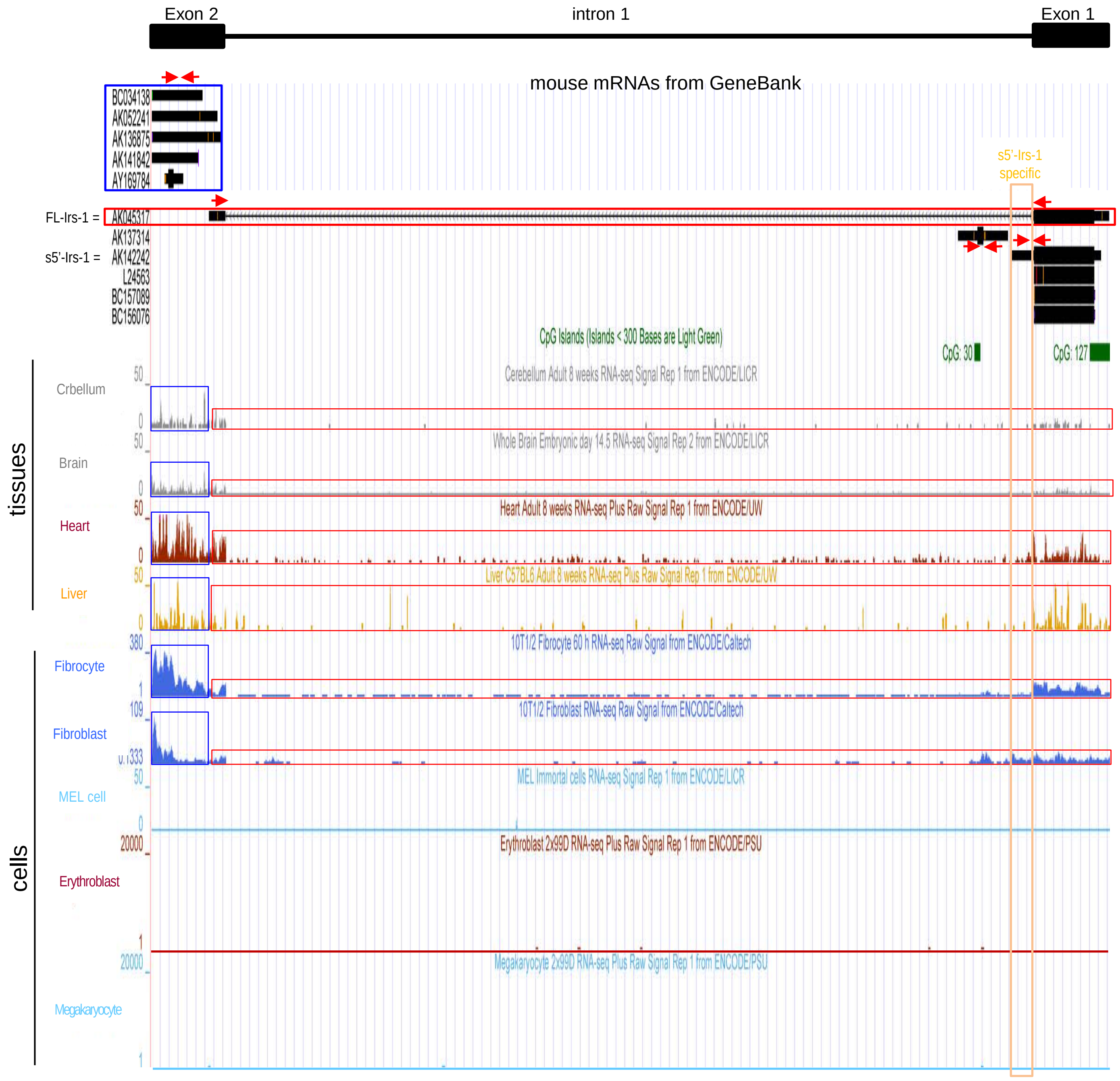

B

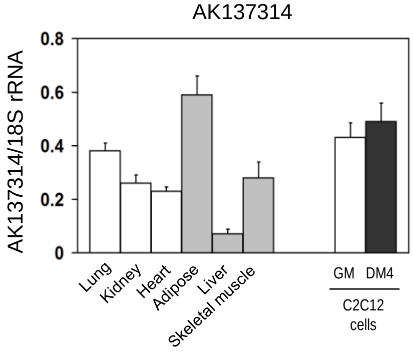

Supplementary Figure S1. Expression of *Irs-1* transcript variants

(A) Representative scheme of *Irs-1* transcript variants in UCSC genome database (mouse mRNAs from GenBank, CpG islands and RNA-Seq signal data). FL-*Irs-1* mRNA is indicated by red squares. Specific region of s5'-*Irs-1* mRNA is indicated by an orange square. Exon 2-derived transcripts are indicated by blue squares. Primer sets for qPCR analysis are shown by red arrows.

(B) Expression levels of AK137314 transcript encoded in the intron 1 in the indicated mouse tissues, proliferating C2C12 myoblasts (GM, growth medium) and differentiating C2C12 myotubes (DM4, differentiation medium for 4 days). Mean ± SD, n=4.
