# Supplementary material for: A novel myogenic function residing in the 5′ non-coding region of Insulin receptor substrate-1 (Irs-1) transcript
Source: BMC Cell Biol. 2015 Mar 11;16:8. doi: 10.1186/s12860-015-0054-8 (PMC4373113; doi:10.1186/s12860-015-0054-8)
Supplement: Additional file 2: Figure S2. — Effect of s5′-Irs-1 transcript on C2C12 differentiation. [file 12860_2015_54_MOESM2_ESM.pdf]

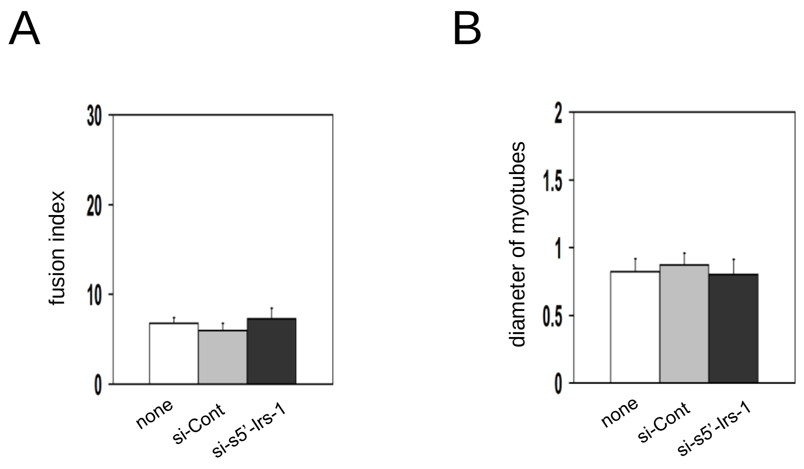


**Supplementary Figure S2. s5'-*Irs-1* transcript does not affect C2C12 differentiation**

Effect of knockdown of s5'-*Irs-1* mRNA on differentiation of C2C12 myoblast. Fusion index (A) and a mean diameter of myotubes (B) are shown. Mean ± SD, n=50, *p < 0.01 *versus* no treatment (none).
