# Supplementary material for: A novel myogenic function residing in the 5′ non-coding region of Insulin receptor substrate-1 (Irs-1) transcript
Source: BMC Cell Biol. 2015 Mar 11;16:8. doi: 10.1186/s12860-015-0054-8 (PMC4373113; doi:10.1186/s12860-015-0054-8)
Supplement: Additional file 3: Figure S3. — Effect of FL-Irs-1 transcript on expression of several myogenic factors. [file 12860_2015_54_MOESM3_ESM.pdf]

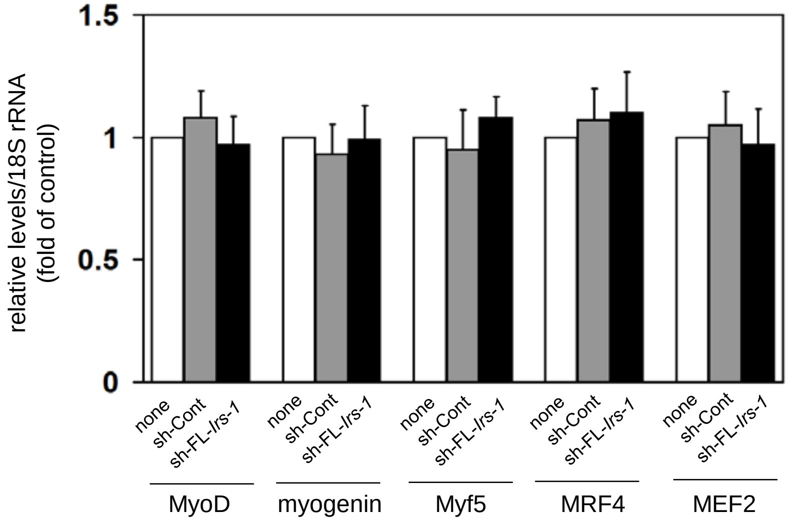

Supplementary Figure S3. Effect of knockdown of FL-*Irs-1* mRNA on expression of myogenic regulators

C2C12 myoblasts were transfected with the sh-FL-*Irs-1* mRNA. Expression levels of endogenous MyoD, myogenin, Myf5, MRF4 and MEF2 were determined by RT-qPCR analysis. Mean ± SD, n=4.
