# Supplementary material for: A novel myogenic function residing in the 5′ non-coding region of Insulin receptor substrate-1 (Irs-1) transcript
Source: BMC Cell Biol. 2015 Mar 11;16:8. doi: 10.1186/s12860-015-0054-8 (PMC4373113; doi:10.1186/s12860-015-0054-8)
Supplement: Additional file 4: Figure S4. — Effect of knockdown of Dicer or Upf-1 on FL-Irs-1 function. [file 12860_2015_54_MOESM4_ESM.pdf]

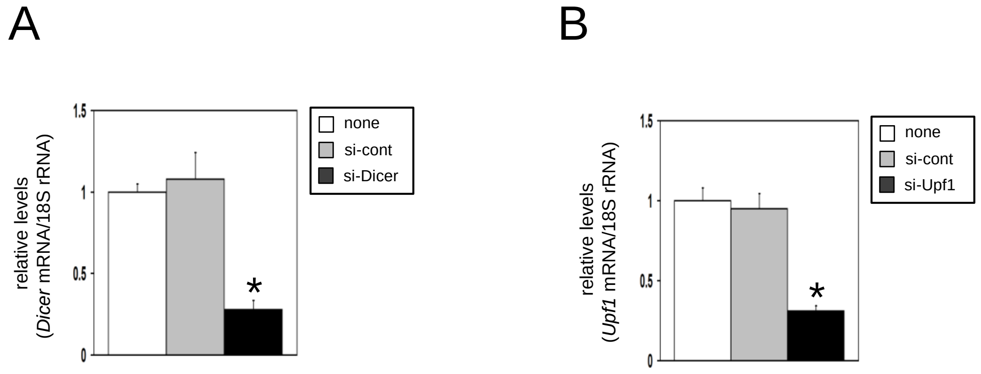

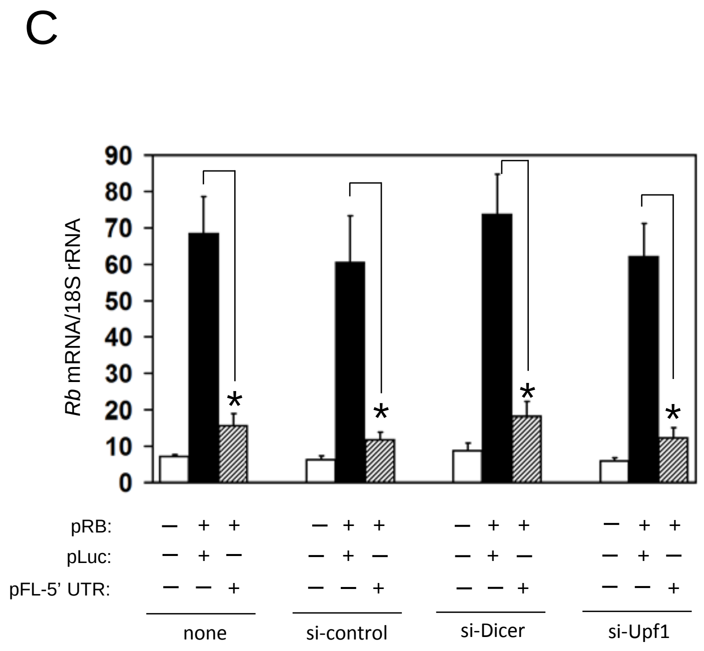

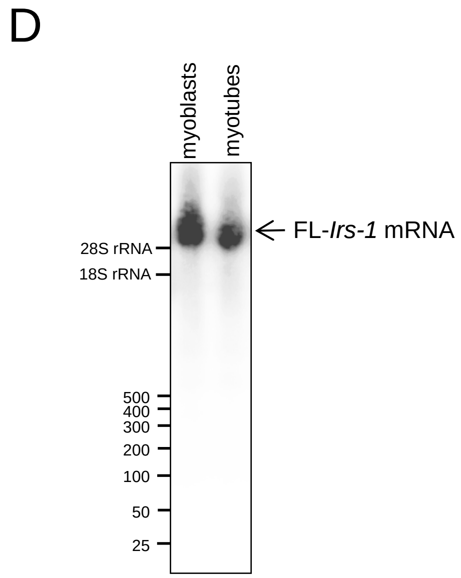

Supplementary Figure S4. Reduction of *Rb* mRNA by FL-*Irs-1* transcript is independent of Dicer and Upf1

(A, B) Knockdown efficiency of Dicer mRNA by si-Dicer (A) or Upf1 mRNA by si-Upf1 (B). Mean ± SD, n=5, *p < 0.01 *versus* no treatment (none)

(C) Effect of knockdown of Dicer or Upf1 on reduction of Rb mRNA induced by the 5'UTR of FL-*Irs-1*. Expression levels of Rb mRNA in C2C12 myoblasts that were co-transfected with plasmids expressing Rb mRNA and FL-*Irs-1* mRNA 5'UTR in combination with the indicated siRNA (si-control, si-Dicer or si-Upf1). Mean ± SD, n=4, *p < 0.01 *versus* control transfectant (pRB + pLuc).

(D) Representative Northern Blot analysis in C2C12 myoblasts and myotubes. Northern hybridization was performed using an LNA-based antisense probe against FL-*Irs-1* 5'UTR (FL-5'-AS-ODN) as shown in Figure 3C.
